# Consolidating Russia and Eurasia Antibiotic Resistance Data for 1992–2014 Using Search Engine

**DOI:** 10.3389/fmicb.2016.00294

**Published:** 2016-03-08

**Authors:** Alexander Bedenkov, Vitaly Shpinev, Nikolay Suvorov, Evgeny Sokolov, Evgeniy Riabenko

**Affiliations:** ^1^Medical Department, AstraZeneca Pharmaceuticals LLCMoscow, Russia; ^2^Yandex Data Factory, Yandex LLCMoscow, Russia

**Keywords:** Russia, antibiotic, antimicrobial, resistance, microorganism, database, algorithm, surveillance

## Abstract

**Background:** The World Health Organization recognizes the antibiotic resistance problem as a major health threat in the twenty first century. The paper describes an effort to fight it undertaken at the verge of two industries—healthcare and Data Science. One of the major difficulties in monitoring antibiotic resistance is low availability of comprehensive research data. Our aim is to develop a nation-wide antibiotic resistance database using Internet search and data processing algorithms using Russian language publications.

**Materials and Methods:** An interdisciplinary team built an intelligent Internet search filter to locate all publicly available research data on antibiotic resistance in Russia and Eurasia countries, extracted it, and collated it for analysis. A database was constructed using data from 850 original studies conducted at 153 locations in 12 countries between 1992 and 2014. The studies contained susceptibility and resistance rates of 156 microorganisms to 157 antibiotic drugs.

**Results:** The applied search methodology was highly robust in that it yielded search precision of 58 vs. 20% in a typical Internet search. It allowed finding and collating within the database the following data items (among many others): publication details including title, source, date, authors, etc.; study details: time period, locations, research organization, therapy area, etc.; microorganisms and antibiotic drugs included in the study along with prevalence values of resistant and susceptible strains, and numbers of isolates. The next stage in project development will try to validate the data by matching it to major benchmark studies; in addition, a panel of experts will be convened to evaluate the outcomes.

**Conclusions:** The work provides a supplementary tool to national surveillance systems in antibiotic resistance, and consolidates fragmented research data available for 12 countries for a period of more than 20 years.

## Background

Antibiotic resistance is resistance of a microorganism to an antibiotic drug that was originally effective for treatment of infections caused by it (World Health Organization, 2015). This resistance becomes an increasingly serious threat to global public health that requires urgent action across all government sectors and society (Boucher et al., 2009; Carlet et al., 2014; Llor and Bjerrum, 2014). Without urgent action, we are heading for the second pre-antibiotic era, in which common infections and minor injuries can once again kill (Livermore and British Society for Antimicrobial Chemotherapy Working Party on The Urgent Need: Regenerating Antibacterial Drug Discovery and Development, 2011; Appelbaum, 2012; Roca et al., 2015). The World Health Organization (WHO)'s 2014 report on global surveillance of antimicrobial resistance revealed high rates of resistance in all regions of the world (World Health Organization, 2014). Yet, only a few countries (34 out of 133 participating in the WHO's survey) have a comprehensive national plan to fight resistance to antibiotics and other antimicrobial medicines. Significant gaps exist in many countries in tracking of antibiotic resistance (World Health Organization, 2014). This paper presents an approach that yields consolidated data for monitoring, and therefore controlling, resistance profiles. The resultant database built on publicly available research data on antibiotic resistance using a combination of a customized Internet search engine and data processing techniques provides a novel instrument for resistance analysis. It can become a supplementary tool for national surveillance systems in some countries, in particular in Russia and Eurasia, consolidating numerous studies of antibiotic resistance undertaken by a number of research centers into a single, nation-wide, picture. Used together, existing and evolving resistance data management approaches can ensure effective surveillance, which can reveal patterns and identify trends and outbreaks.

The problem of microorganisms' resistance to drugs has spread worldwide and is acute in many countries. Knowing susceptibility of particular microorganisms to antibiotics is crucial for their rational prescription that, in turn, leads to successful treatment of diseases. This is what patients, doctors, pharmaceutical companies, and society alike, wish to achieve. National antibiotic resistance surveillance systems in Russia and Eurasia are still taking their first steps toward maturity. One of the major difficulties in monitoring resistance profiles is low availability of comprehensive data on antibiotic resistance coming from various research studies. Such data are highly fragmented and not being applied systemically. Only a handful of top microbiology research centers provide a clear view on the results of their studies by regularly publishing reports in Russian and Eurasian national journals. Studies of other research groups remain virtually unnoticed, although they provide a valuable source of antibiotic resistance data derived from day-to-day practice. Ideally, all available records should be merged together within a single database that will most certainly prove to be invaluable for the national healthcare system as the source of real evidence on antibiotics resistance. This is where the interdisciplinary project undertaken at the verge of two industries: healthcare, presented by AstraZeneca, and data science, presented by Yandex Data Factory, can help national surveillance systems of Russia and Eurasia to evolve.

Inspired by the fact that the Internet has become the megastore of various data, and rapid evolution of data processing technologies, a hypothesis was developed that some new useful knowledge for the national surveillance system can emerge from readily available data through the combination of Internet search and data processing algorithms. It implied that fragmented research studies on antimicrobial resistance published in the Russian language on the Internet could be successfully discovered and collated into a nation-wide antibiotic resistance database. Only publicly available research data not protected by copyright or other rights would be considered for inclusion. Such a database would not only allow to view the current perspective, but also to see the dynamics of pathogenic microorganisms and—if coupled with antibiotics consumption data—could establish a basis for identifying trends and making forecasts. The approach presented here complements previous attempts (Harbarth and Emonet, 2006; Brandt et al., 2014) to use the Internet for gathering data on antibiotic resistance, yet its novelty is in (a) retrieving data from the Internet not limited by particular websites, (b) storing it in the database for subsequent use, and (c) targeting materials in the Russian language.

## Results and discussion

The Internet search filter designed for the project reduced the working set of documents from more than 10^12^ to nearly 60,000. Still, this provided a very suboptimal filtering algorithm demanding further refinements in identifying relevant documents from the entire pool. In order to significantly improve quality, the search model was optimized by three consecutive iterations of machine learning. At the start of each iteration, a group of assessors manually classified top documents from the list ranked by the current model, defining their relevance and type. These classifications were then fed into the search model, and the relevance score for each document was recalculated. After each iteration, the percentage of relevant documents in the first 1000 positions improved significantly, growing from the original 2% to more than 58% eventually. Altogether, assessors classified 4385 documents to reach this level. As an outcome of applying the refined search algorithm, a list of URLs was produced, ordered by the underlying document relevance score in the descending order.

While at the start of the project the target search precision of 20% was set (Robertson and Hull, 2001), it was actually possible to exceed this target significantly. Thanks to machine learning with the help of manual document classification, the search algorithm provided precision of 58% for the top 1000 documents.

At first, consideration was given to automatic document parsing and data extraction. An experiment was carried out using a sample set of 50 documents, with the aim of determining the technical capability of automatically identifying and extracting target datasets from the documents. It was observed that datasets were represented in a diverse variety of styles: tables, charts, histograms and—unsurprisingly—free text. In particular, 60% of sample documents contained table data, 20%—free text, and the remaining 20%—charts or histograms. Within table format, nearly one third of tables had microorganism names listed in headers, another third—in rows, and the rest—in table captions or adjacent text. The most difficult format for data parsing, though, was charts and histograms as it required an OCR system able to extract numeric data from graphics. Adding to the complexity were abbreviations and various formats of names and numbers. The conclusion derived from the experiment was that wide variety of data representation formats required a highly complicated automatic parsing algorithm that would adapt its fact extraction model to virtually every document.

In parallel with this, manual document screening was under way to identify relevant documents in the set of 58,821 documents found automatically. As at some point the number of relevant documents in the set was evaluated slightly below 1000, the option of manual data extraction was chosen. Automatic data extraction and transformation to a single format was not justified in our case and would be deemed reasonable only for volumes of documents a few orders of magnitude higher.

**Figure 1:**
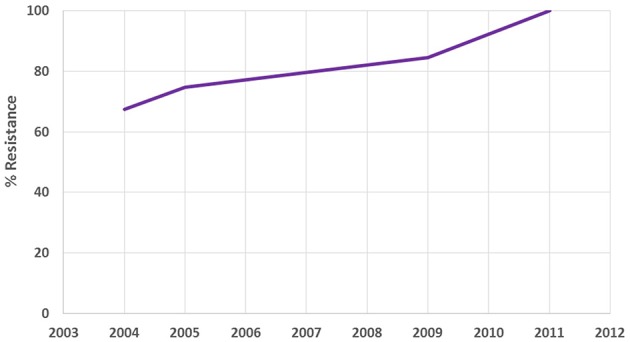
**Sample antibiotic resistance data from the city of Yakutsk (Russia)**. The chart shows resistance of *Pseudomonas aeruginosa* to Gentamicin.

**Figure 2:**
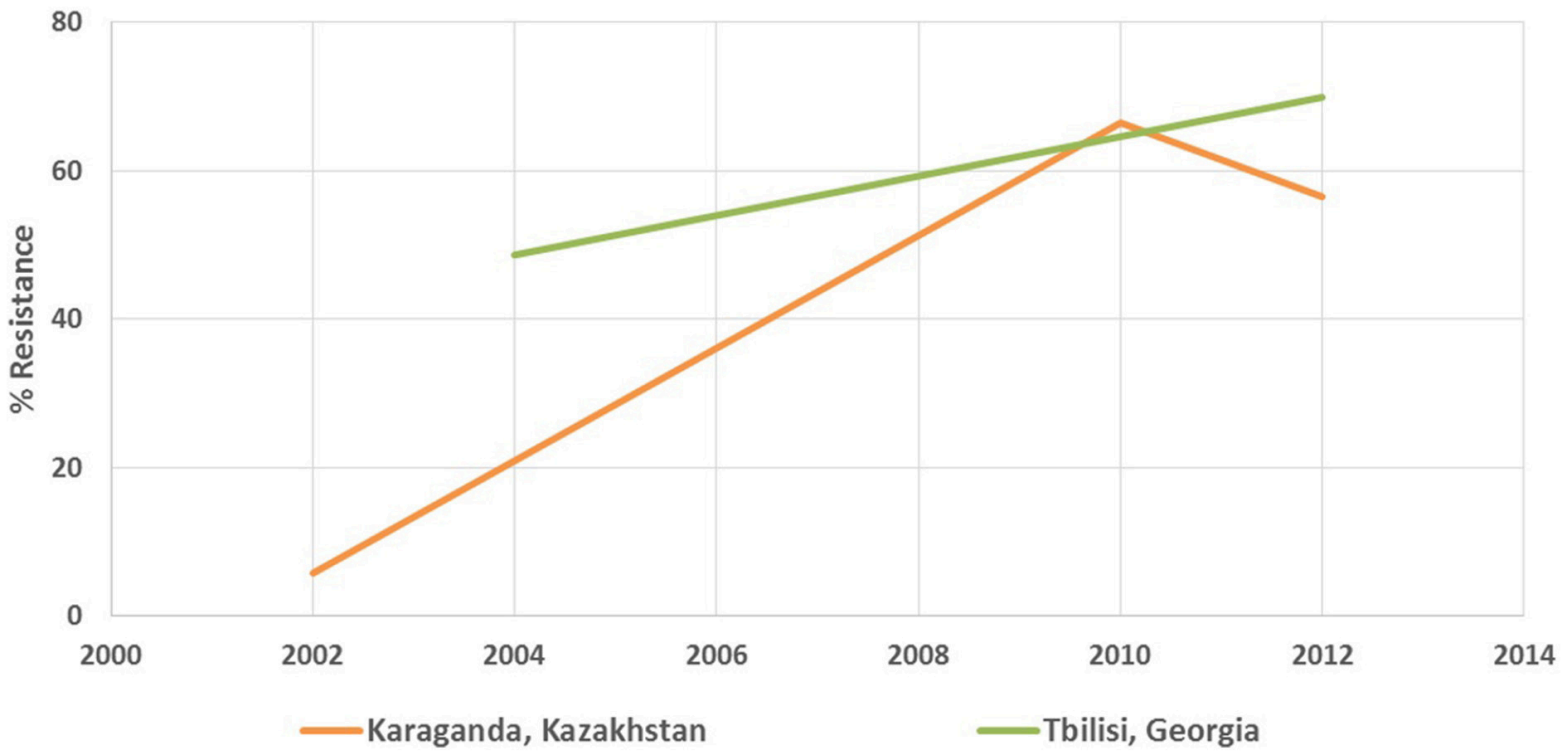
**Sample antibiotic resistance data from the cities of Karaganda (Kazakhstan) and Tbilisi (Georgia)**. The chart shows resistance of *Pseudomonas aeruginosa* to Ciprofloxacin.

The antimicrobial resistance database was designed and developed specifically for the project using one of popular relational database management systems. The data management team extracted resistance data from documents and stored it in the database for further analysis. Eight hundred and fifty original studies were collated to have been published in the period between 1992 and 2014, containing susceptibility and resistance rates of 156 microorganisms to 157 antibiotic drugs. The studies were conducted in 153 locations of 12 countries. While the project targeted only Russian language studies on antibiotic resistance, multiple publications in Russian from several Eurasia countries—Georgia, Kazakhstan, Uzbekistan, etc.—were also discovered during its course. Figures 1, 2 contain sample data.

The project confirmed the fact that studies conducted in Russia use different interpretive criteria and breakpoints, e.g., EUCAST, CLSI, and local Russian guidelines. For instance, the share of NCCLS/CLSI-compliant studies reaches 27% in the resultant database. For correct stratification of data in each susceptibility category, a reference to interpretive criteria was stored for each study. At the same time however, the database currently has no feature to account for different susceptibility rules used in different years.

Another limitation of the current database is that is lacks a simple way of distinguishing between community-acquired and hospital-associated infections. Currently, this can be achieved using a rather complex set of attributes. Further modifications of the database will make it possible to differentiate these two types of infection easily.

**Figure 3:**
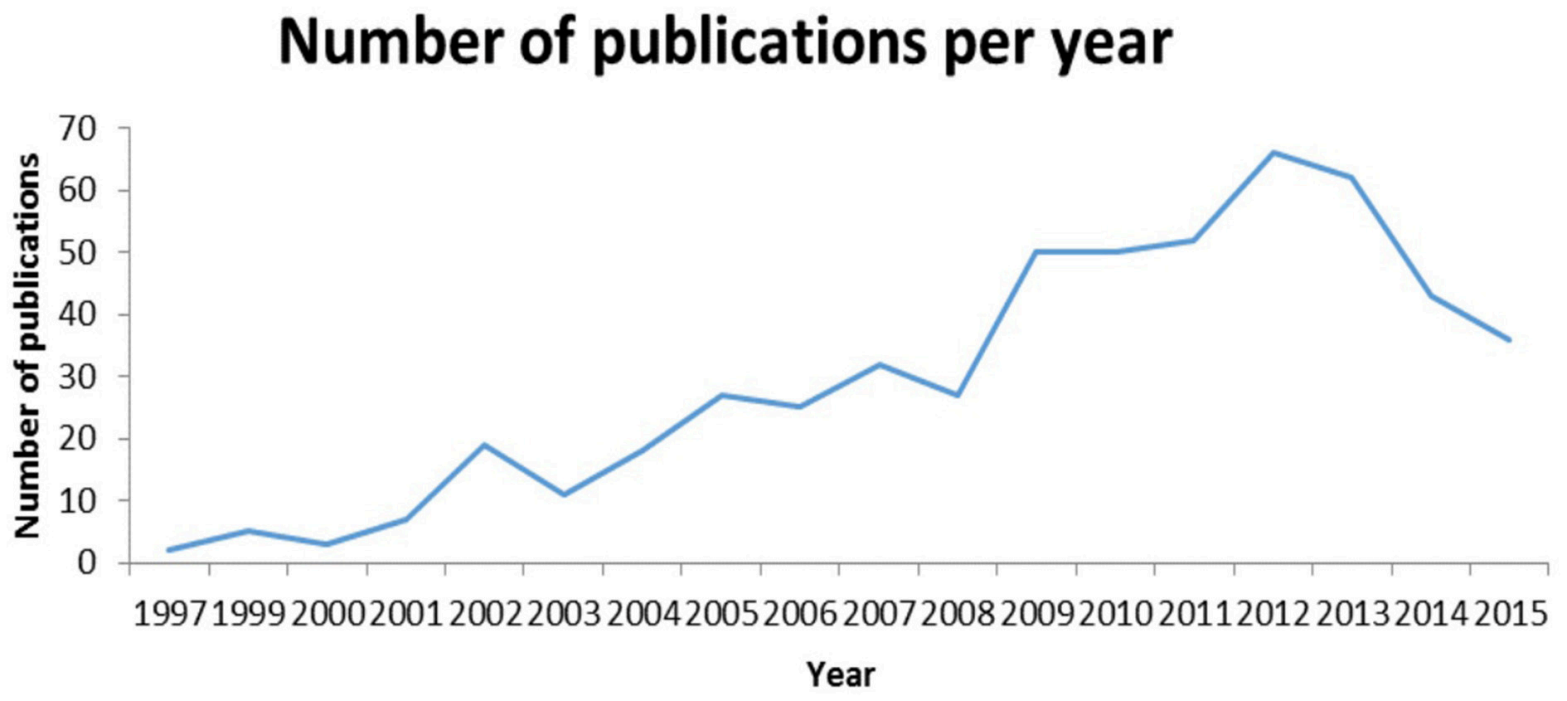
**Statistics on publications on antimicrobial resistance in Russia**. The dynamics of number of publications is clearly linear. The downfall in 2014–2015 is likely due to the typical lifecycle of a study in which 1–2 years elapse between the study and publication.

An interesting by-product of the project was the statistics on yearly number of publications in the subject matter area. In the last 5 years, the number of new articles fluctuated in the range of 50–70 per year; see Figure 3 for details.

The next stage in project development will attempt to validate the collected data by matching it against some major benchmark studies, and establish Advisory Boards of experts to evaluate the outcomes.

In its current format, the output of the project can only be used by the Russian-speaking audience. However, with moderate efforts, it can be converted to English thus making it accessible by much wider audience.

The project has shown that it can be instrumental in creating a holistic antibiotic resistance surveillance framework. Its main value is in discovering and consolidating multiple fractions of data that in many instances are left outside the mainstream analysis. The presented approach by no means purports to be the universal solution; it merely serves to provide a supplementary tool. Its shortcomings are obvious: uncertain set of source data, sizeable number of documents to screen for relevance, and laborious data extraction. Further interpretation of collected data needs to be mindful of the fact that quality of studies and underlying data was accepted “as is” and not questioned.

## Conclusions

It appears prudent to combine outcomes of this project with other approaches and research tools in order to create an integrated picture of antibiotic resistance at national level including also the epidemiology outlook. In particular, its extensive database can be one of the cornerstones on which data management system for the surveillance framework is built. It will be of great worth if such a system is established in Russia and Eurasia countries and populated with the most up-to-date research data provided by laboratories and research centers in order to successfully combat antibiotic resistance.

## Materials and methods

### Search inputs

Initially, a group of medical experts formed a list of keywords and names, carefully selecting those pertaining to the topic (Table 1). The second list, containing non-inclusive words—e.g., virus, fungus—was composed to exclude documents seemingly relevant yet belonging to a different subject. This, in part, helped set an important constraint in the algorithm, namely, to consider as irrelevant all non-original publications, e.g., reviews, clinical recommendations, articles on treating infectious diseases, etc. Another input to the search model was the target document structure for which to search, as it was observed that relevant materials are often embedded within a complex document, e.g., a volume of articles or a conference book.

**Table 1:** **List of search keywords**.

**Antibiotic names**	**Microorganism names**	**General keywords**
Avibact	Acinetobacter	Antimicrobial
Amikacin	Aeruginosa	Antibiotic
Amoxicillin	Aureus	Resistance
Ampicillin	Baumannii	Susceptibility
Aztreon	Burkholderia	
Azithromycin	Campylobacter	
Bedaquiline	Catarrhalis	
Benzylpenicillin	Cepacia	
Cefaclor	Clostridium	
Cefadrox	Coli	
Cephalexin	Corynebacterium	
Cefazolin	Difficile	
Cefep	Enterobacter	
Cefix	Enterobacteriaceae	
Cefotax	Enterococcus	
Cefoxitin	Escherichia	
Cefpodox	Faecalis	
Ceftaroline	Faecium	
Ceftazid	Flavobacter	
Ceftobiprol	Flexneri	
Ceftriaxone	Gonorrhea	
Cefurox	Haemophilus	
Chloramphenicol	Helicobacter	
Ciprofloxacin	Influenza	
Clarithromycin	Jejuni	
Clavulanic acid	Klebsiella	
Clindamycin	Listeria	
Cloxacillin	Maltophilia	
Colistin	Meningitis	
Dalbavancin	Mirabilis	
Daptomycin	Monocytogenes	
Delamanid	Moraxella	
Dicloxacillin	Multocida	
Doripen	Mycobacterium	
Doxycycline	Neisseria	
Erythromycin	Oxytoca	
Ertapen	Pasteurella	
Fidaxomicin	Pneumoniae	
Flucloxacillin	Proteus	
Furazidin	Pseudomonas	
Gentamicin	Pylori	
Imipen	Pyogenes	
Levofloxacin	Salmonella	
Linezolid	Serratia	
Mecillinam	Shigella	
Meropen	Staphylococcus	
Metronidazol	Stenotrophomonas	
Minocycline	Streptococcus	
Moxifloxacin	Tuberculosis	
Mupirocin		
Nalidix		
Netilmicin		
Nitrofurantoin		
Norfloxacin		
Ofloxacin		
Oritavancin		
Oxacillin		
Pefloxacin		
Phenoxymethylpenicillin		
Phosphomycin		
Piperacillin		
Rifampicin		
Roxythromycin		
Spectinomycin		
Telavancin		
Telithromycin		
Tetracycline		
Teicoplanin		
Ticarcillin		
Tedizolid		
Tigecycline		
Tobramycin		
Trimetopr		
Vancomycin		

*Some antibiotic and microorganism names are intentionally truncated*.

The source data came from the Yandex content system, a distributed data store keeping all freely accessible Internet pages scanned by the Yandex web crawler. Once a document is loaded in the content system, it undergoes several stages of processing during which a multitude of numerical features and statistics of the document are calculated and included in the search index—the primary data structure enabling keyword search. The search engine and index were provided by Yandex Data Factory, the machine learning and data analytics experts who use data science to meet particular business needs involving working with large amounts of data, by building upon the advanced technologies of their parent company, Yandex.

### Search model

Having received the search requirements, a group of software engineers and data scientists translated them into a set of search rules, included in the search model, and applied to the search index. The search model suggested that relevant documents are a subset of N documents with highest relevance scores. The value of N is arbitrarily chosen depending on desired levels of search precision and recall that are the main characteristics of search quality. They are defined as follows:

- **Search precision** is the proportion of documents in the selected subset that are relevant.
- **Search recall** is the proportion of all the relevant documents that are selected.

A few groups of document features were calculated in order to determine document relevance:

1. Vector Space Model (Salton et al., 1975) representation of the text (i.e., counts of all possible words);
2. features based on word embedding (Mikolov et al., 2013; Le and Mikolov, 2014);
3. website from where the document was downloaded;
4. document features used in Yandex ranking algorithm;
5. counts of keywords from relevant and non-inclusive lists;
6. positional properties of relevant keywords such as a difference between first and last occurrence of such keywords.

Using these features as the basis, machine learning was applied to train the classification model to automatically screen documents as relevant or irrelevant. The machine learning toolset was based on MatrixNet—Yandex's implementation of gradient boosting over oblivious decision trees. The resulting model calculates the relevance score for each document—the measure of its confidence in the document's belonging to the positive (or relevant) class.

### Quality metrics

**Figure 4:**
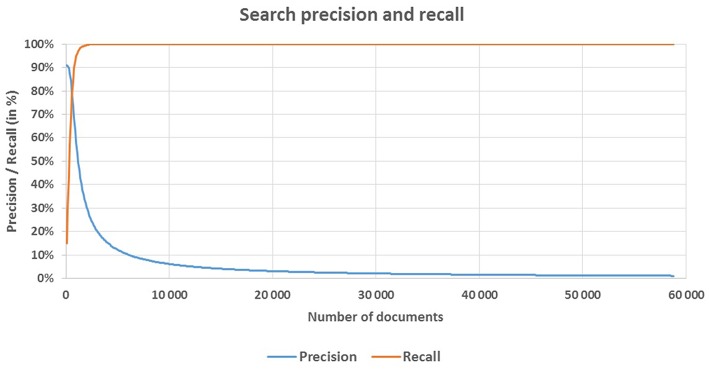
**Search precision and recall**. Density of relevant documents in the set sorted by the relevance score decreases as the size of the set grows bigger. The more relevant documents are placed in the first thousand documents, the less are found in the second, etc. Therefore, search precision decreases monotonically toward the end of the selected set. For our task, this implies that only the first few thousand documents need to be analyzed where the density of relevant documents is nearing maximum. Nearly 99% of relevant documents are discovered within the first 2000 documents.

Search quality metrics at *N* = 1000 are quite illustrative; of the first 1000 documents, 636 were classified by assessors. Precision at *N* = 1000 equals 57.9%, and the recall is 94.8%. Precision of 50% is reached at *N* = 1186, with recall of 97.1%. Precision of 80% is reached at *N* = 592, with recall of 77.6%. Maximum level of recall (100%) is reached at *N* = 2307, at which point the precision is only 26.5%. With *N* = 611, precision and recall are leveled and equal to 78.4%. Figure 4 shows the precision and recall curves depending on the number (N) of selected documents.

### Data extraction

Once relevant documents were identified, the data extraction stage began. The search model was created on the premise that relevant documents contain—whether in table or free text form—basic datasets of antibiotic resistance data. Datasets were bound to include the following mandatory items: antibiotic drug name; microorganism; measures of resistance and susceptibility; year of study; city or territory of study; and number of samples studied. As the option of manual data extraction was chosen, a data management team was formed and trained in order to populate the antimicrobial resistance database. Throughout the process of collating the data in the database, various automatic and manual data checks and validations were applied to ensure data quality.

## Author contributions

AB participated in the conception and design of the study, and the analysis of data. VS participated in the analysis of data, and drafting/providing critical revision of the article. NS participated in the analysis of data. ES and ER participated in the acquisition and analysis of data. All authors read and approved the final manuscript.

## Funding

The study was funded by AstraZeneca Pharmaceuticals LLC.

### Conflict of interest statement

The authors declare that the research was conducted in the absence of any commercial or financial relationships that could be construed as a potential conflict of interest. AB, NS, and VS are employees of AstraZeneca. ES and ER are employees of Yandex. None of the authors owns any stock or shares in AstraZeneca and/or Yandex.

## References

[B1] Appelbaum P. C. (2012). 2012 and beyond: potential for the start of a second pre-antibiotic era? J. Antimicrob. Chemother. 67, 2062–2068. 10.1093/jac/dks213 22687888

[B2] Boucher H. W. Talbot G. H. Bradley J. S. Edwards J. E. Gilbert D. Rice L. B.. (2009). Bad bugs, no drugs: no ESKAPE! An update from the Infectious Diseases Society of America. Clin. Infect. Dis. 48, 1–12. 10.1086/595011 19035777

[B3] Brandt C. Makarewicz O. Fischer T. Stein C. Pfeifer Y. Werner G.. (2014). The bigger picture: the history of antibiotics and antimicrobial resistance displayed by scientometric data. Int. J. Antimicrob. Agents 44, 424–430. 10.1016/j.ijantimicag.2014.08.001 25216545

[B4] Carlet J. Pulcini C. Piddock L. J. (2014). Antibiotic resistance: a geopolitical issue. Clin. Microbiol. Infect. 20, 949–953. 10.1111/1469-0691.12767 25040923

[B5] Harbarth S. Emonet S. (2006). Navigating the World Wide Web in search of resources on antimicrobial resistance. Clin. Infect. Dis. 43, 72–78. 10.1086/504877 16758421

[B6] Le Q. Mikolov T. (2014). Distributed representations of sentences and documents, in Proceedings of the 31st International Conference on Machine Learning (Beijing), 1188–1196.

[B7] Livermore D. M. British Society for Antimicrobial Chemotherapy Working Party on The Urgent Need: Regenerating Antibacterial Drug Discovery Development (2011). Discovery research: the scientific challenge of finding new antibiotics. J. Antimicrob. Chemother. 66, 1941–1944. 10.1093/jac/dkr262 21700626

[B8] Llor C. Bjerrum L. (2014). Antimicrobial resistance: risk associated with antibiotic overuse and initiatives to reduce the problem. Ther. Adv. Drug Saf. 5, 229–241. 10.1177/2042098614554919 25436105 PMC4232501

[B9] Mikolov T. Chen K. Corrado G. Dean J. (2013). Efficient estimation of word representations in vector space, in Proceedings of Workshop at ICLR, arXiv:1301.3781v1.

[B10] Robertson S. Hull D. A. (2001). The TREC-9 Filtering Track Final Report. Available online at: http://trec.nist.gov/pubs/trec9/papers/filtering_new.pdf (Accessed October 16, 2015).

[B11] Roca I. Akova M. Baquero F. Carlet J. Cavaleri M. Coenen S.. (2015). The global threat of antimicrobial resistance: science for intervention. New Microb. New Infect. 6, 22–29. 10.1016/j.nmni.2015.02.007 26029375 PMC4446399

[B12] Salton G. Wong A. Yang C. S. (1975). A vector space model for automatic indexing. Commun. ACM 18, 613–620.

[B13] World Health Organization (2014). Antimicrobial Resistance: Global Report on Surveillance 2014. Available online at: http://www.who.int/drugresistance/documents/surveillancereport/en/ (Accessed October 16, 2015).

[B14] World Health Organization (2015). Antimicrobial Resistance (No. 194) [Fact sheet]. Available online at: http://www.who.int/mediacentre/factsheets/fs194/en/

